# Croup Treated by Sulphur

**Published:** 1869-02

**Authors:** 


Croup Treated by Sulphur.—M. Lagauterie (*Half-Yearly Abstract*) gives in croup, teaspoonful doses, every hour, of a mixture of sulphur and water (a teaspoonful to a glass of water), with effects which he describes as wonderful. The cure, in seven very severe cases, was accomplished in two days, the only symptom remaining being a slight cough. An observation of the effect of sulphur on the oidium of vines, led to its use in croup.

